# Parrotfish demonstrate little behavioural shift during marine heatwaves

**DOI:** 10.1098/rsbl.2026.0337

**Published:** 2026-09-09

**Authors:** Dan Bez Golanski, Renanel Pickholtz, Moshe Kiflawi, Glenn T. Crossin, Gitai Yahel, Jonathan Belmaker

**Affiliations:** ^1^ School of Zoology, Faculty of Life Sciences, Tel Aviv University, Tel Aviv-Yafo, Israel; ^2^ Faculty of Marine Sciences, Ruppin Academic Center, Hefer Valley, Israel; ^3^ Interuniversity Institute for Marine Sciences in Eilat, Eilat, Israel; ^4^ Department of Life Sciences, Ben-Gurion University of the Negev, Beer-Sheva, Israel; ^5^ Department of Biology, Dalhousie University, Halifax, Nova Scotia, Canada; ^6^ The Steinhardt Museum of Natural History, Tel Aviv University, Tel-Aviv, Israel

**Keywords:** thermal stress, climate change, extreme climate events, coral reef, behavioural ecology

## Abstract

The world is experiencing notable increases in the duration, intensity and frequency of marine heatwaves (MHWs). Documented effects of MHWs on fishes encompass various population- and community-level impacts, ranging from localized mass mortalities to transient or semi-permanent range shifts, while some populations show little measurable change. However, there is limited information on *in situ* behavioural responses of individuals before, during and after MHWs. In this study, we used acoustic telemetry to examine the activity levels, depths and daily displacement of 32 terminal-phase male parrotfishes belonging to five species across 12 moderate-sized MHWs (2016–2021). The study took place in the northern Red Sea and coincided with a mass mortality of parrotfishes during a rapid warming event. The parrotfishes exhibited only minuscule behavioural changes during or after the MHWs, regardless of how the MHW was defined or characterized. Thus, within the range of MHWs studied, MHWs have little to no impact on parrotfishes’ behaviour. Our observations suggest that either the mechanisms by which parrotfishes respond to thermal stress are not behavioural, or that such responses may only occur under more severe MHWs.

## 1. Introduction

A growing concern in marine ecosystems is the prevalence of marine heatwaves (MHWs) [1,2], defined as prolonged, discrete periods of anomalously warm seawater temperatures [3]. These events have been associated with a variety of outcomes, including mass mortalities, species range shifts and changes in community structure [4–10]. Although numerous studies have examined the impact of MHWs on fishes, most focus on population- or community-level effects [11–15], often yielding inconsistent results [15–21]. Individual-level studies are largely confined to laboratory settings, focusing on molecular or physiological responses [22–26]. Thus, a critical gap remains regarding fish behavioural responses to MHWs in nature [27,28], necessitating continuous *in situ* data to understand how individual behavioural shifts drive higher level MHW-driven ecological changes.

In ectothermic organisms, elevated temperatures for periods of days to weeks, such as those caused by MHWs, accelerate metabolic rates [22,23,29,30] and may create a mismatch between energy intake and demand, potentially reducing fitness [23,31–34]. Consequently, behavioural modifications may serve as a coping mechanism against thermal fluctuations [29]. Fishes may adjust their depth distributions by seeking thermal refugia in deeper, cooler waters [35–38]. Such a change can carry fitness costs, such as reduced access to foraging grounds or different prey availability. Similarly, altering activity levels [39], either by increasing foraging to meet elevated energy demands or by reducing activity to conserve energy, imposes its own energetic costs and potential fitness trade-offs [40,41]. The extent to which wild fishes employ these compensatory strategies during short-term thermal extremes remains unclear.

Parrotfishes (sub-family Scarinae) play a vital role in coral reef ecosystems by controlling algal growth, maintaining coral dominance, and hence supporting the reef's overall health [42]. A mass mortality event in the northern Red Sea suggests that this clade is highly vulnerable to rapid increases in seawater temperature [5]. Here, using 5 years of acoustic tracking, we examine shifts in depth, activity and movement of 32 parrotfishes from five species in response to MHWs. These data provide a rare opportunity to examine individual-level behavioural changes associated with MHWs.

## 2. Methods

### (a) Study site

The Gulf of Aqaba is a semi-enclosed basin located in the northern Red Sea. It is characterized by a monthly average sea surface temperature (SST) ranging between 21°C in winter and 27°C in summer [43]. Regional SST is increasing at a rate of 0.5°C per decade [43], more than twice the global average [44]. This warming coincides with an increase in duration, intensity and frequency (>1.5 events decade^−1^) of MHWs [45,46].

**Figure 1.**
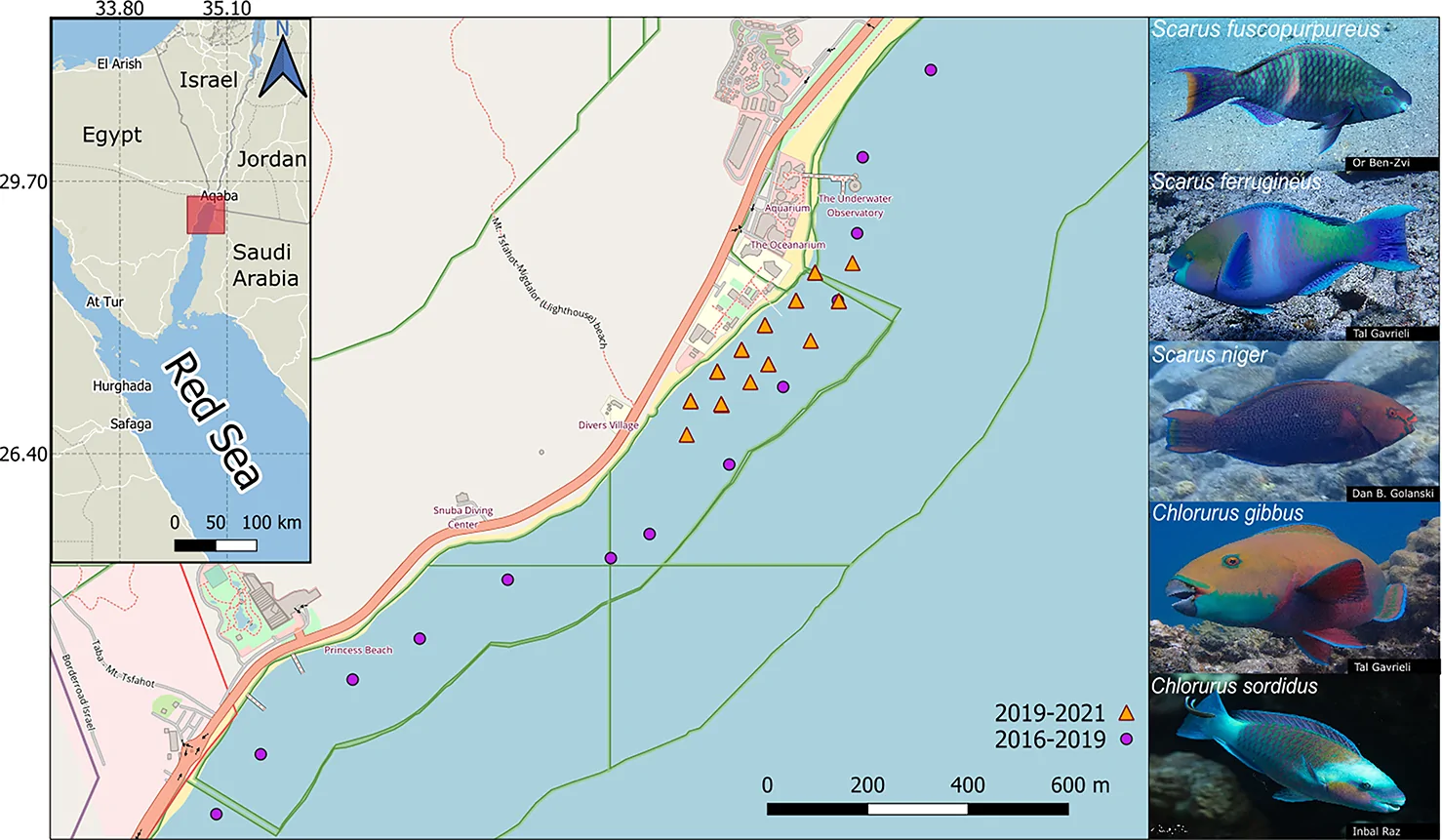
Map of the study area along the northern Gulf of Aqaba, Red Sea. The left insert depicts the acoustic array location within the region. Purple circles and orange triangles denote the acoustic receiver's locations. The images on the right represent the studied parrotfish species: *Scarus fuscopurpureus*, *Scarus ferrugineus*, *Scarus niger*, *Chlorurus gibbus* and *Chlorurus sordidus* (top to bottom).

Acoustic telemetry was used to study parrotfishes’ behaviour in response to MHWs; this approach relies on fixed underwater receivers that record coded pulses from small acoustic transmitters. Two acoustic arrays were deployed consecutively in the Red Sea (29°30′ N 34°55′ E; figure 1). The first array (July 2016–February 2019) consisted of 13 underwater receivers (VR2W-69 kHz; Innovasea) arranged in a linear formation parallel to the coastline. This array spanned a 1600 m coastal belt, with overlapping detection ranges to ensure detection reliability. The second array (June 2019–January 2021) consisted of 13 underwater receivers (TBR-700, Thelma Biotel) arranged in two overlapping lines. This array covered 500 m of the northern part of the previous array's extent. The receivers were placed along the reef slope with a detection range extending beyond 40 m in depth. This configuration ensured comprehensive coverage, capturing potential vertical displacements from shallow habitats, where parrotfishes are typically observed [47], down to deeper potential refuges.

### (b) Acoustic data and fish tagging

We analysed acoustic data of 32 terminal-phase male parrotfishes (total length: 28.5–52 cm) that experienced at least one MHW event. The tracked species included purple-brown parrotfish (*Scarus fuscopurpureus*), rusty parrotfish (*Scarus ferrugineus*), dusky parrotfish (*Scarus niger*), heavybeak parrotfish (*Chlorurus gibbus*) and daisy parrotfish *(Chlorurus sordidus*; figure 1).

Fishes were tracked using surgically implanted acoustic transmitters equipped with a pressure sensor and/or a tri-axial accelerometer. To balance high-resolution data collection with extended tracking duration, transmitters were programmed to change from a high rate (1-min interval) for the first 70 days to a lower rate (5-min interval) for the remainder of battery life (approx. 15 months). To minimize physiological or behavioural effects, all implanted transmitters adhered to the transmitter-to-fish mass ratio of 0.02 [48].

We examined three behavioural metrics derived from acoustic tracking data. The first metric, activity level, utilized transmitter-measured tri-axial acceleration, averaged across the three axes at each detection (5 s, at 5Hz), a common proxy for individual activity [49–51]. The second metric was fish depth, derived from the transmitter's instantaneous pressure readings. The third metric, maximum daily displacement, was derived from a positional estimate termed ‘longshore position’. Owing to the spatial configuration required for this calculation, this metric was limited to data collected during the first acoustic array period. For each individual, detections were grouped within a moving window dynamically adjusted to the transmission rate (5 min for 1-min pings; 35 min for 5-min pings) to ensure sufficient spatial averaging. Within each window, we assigned weights to receivers based on the proportion of detections they recorded. We then multiplied these weights by the linear distance of each receiver from the northernmost receiver and summed across all receivers, yielding a weighted average distance. This provided a continuous proxy for an individual's position along the linear acoustic array, with lower values corresponding to the northern end and higher values to the southern end. Maximum daily displacement was defined for each individual as the range between the 5th and 95th percentiles of daily longshore positions. This inter-decile approach filters out anomalous and false acoustic detections. Comprehensive details regarding fish capture, transmitter details, tagging procedures, array configuration, detection range and calculation protocols are provided in [47].

### (c) Defining marine heatwaves

Recent discussion within the scientific community has highlighted how methodological choices in defining MHWs can lead to divergent outcomes. These choices include the selection of baseline periods and whether long-term warming trends are incorporated [52]. However, it remains unclear which approach is most appropriate for assessing organismal responses [53]. We compare two different MHW definitions: a ‘fixed baseline’, which accounts for the long-term warming, and a ‘detrended baseline’, which better represents extreme events relative to current conditions.

We used NOAA's ¼° Daily Optimum Interpolation Sea Surface Temperature dataset [54] to define MHWs. To validate its suitability, we compared it with an independent dataset collected at the Interuniversity Institute for Marine Sciences of Eilat [55], which has been recording measurements 5 days a week since 1988, with missing values interpolated linearly. A strong correlation between the two datasets (*R*² = 0.91) confirmed their comparability. To ensure that observed behavioural shifts are attributed explicitly to MHWs and not to seasonal variations [56], we limited our analysis to MHW events lasting no more than one month.

Fixed-baseline MHWs were defined as periods of at least 5 consecutive days where daily temperatures exceeded the 90th percentile of the climatological baseline, following Hobday *et al.* [3]. For the detrended-baseline MHWs, we applied the method described by Jacox *et al.* [57], which linearly detrends daily SST anomalies before MHW detection (accounting for the long-term warming signal, creating a warming-adjusted climatology), based on the same criteria as [3]. The climatology for defining MHWs was calculated over 30 years (1981–2011). Both approaches were implemented via the *heatwaveR* package [58]. Although the detected MHWs differed between the two definitions in their timing, frequency and duration (supplementary material, tables S1 and S7); behavioural patterns revealed similar patterns. Therefore, the main text includes only the fixed-baseline results, which yielded the highest number of detected MHWs (see supplementary material for the detrended-baseline results).

We compared behavioural metrics across three temporal stages: ‘before’, ‘during (MHW)’ and ‘after’. While the definition of an MHW itself is well-established, delineating the boundaries of the ‘before’ and ‘after’ periods is more subjective. To address this, we defined the ‘before’ and ‘after’ periods as a standardized one-week (7 days) window preceding and following each event. To prevent overlap between temporally proximate MHWs, we truncated each ‘after’ period to end no later than the day before the next MHW, and each ‘before’ period to start no earlier than the day after a preceding MHW. Sensitivity analyses using alternative temporal windows and excluding temporal proximate MHWs yielded consistent patterns with minor exceptions (see supplementary material).

To quantify the strength of the MHWs, we used the following characteristics: mean intensity (average anomaly above the baseline (°C)), maximum intensity (peak anomaly (°C)), onset rate (rate of warming until the peak (°C day^−1^)), maximum absolute intensity (highest observed temperature (°C)), duration (consecutive days above the threshold (days)) and cumulative intensity (sum of daily intensity anomalies (°C days)) as defined by [3].

Using the fixed baseline approach, we identified 12 MHWs for which appropriate acoustic data were available. These events had a duration of 16.6 ± 5.8 days (mean ± 95% CI), with absolute maximum temperatures ranging from 22.0 to 29.4°C. The highest recorded maximum intensity was 2.1°C, and the highest cumulative intensity reached 48.0°C days (supplementary material, figures S1 and S2). A comprehensive breakdown detailing exactly how many MHW each fish experienced and which individuals were used in each analysis is provided in supplementary material, table S2.

### (d) Data analysis

#### (i) Overall mean behavioural response

We started by comparing mean depth, activity level and maximum daily displacement across the three MHW stages. To ensure robust temporal coverage and minimize false detections, acoustic data were filtered to exclude 1-h intervals with fewer than two detections. To ensure reliable, balanced sampling, we retained individual-MHW combinations with ≥50 h of data per MHW stage (or >50 detections per day for displacement), including only individuals with sufficient data across all three stages. Analyses of detection gaps confirmed no significant detection bias across thermal stages (ANOVA, *p* = 0.14). Mean depth, activity and displacement were calculated per individual for each stage in each specific MHW event. The final sample sizes were 56, 45 and 12 fish–MHW combinations for activity (26 individuals), depth (25) and displacement (10), respectively. Notably, several fishes were tracked across multiple events.

MHW impacts on these behavioural metrics were evaluated using generalized linear mixed models (GLMMs) and model selection via the *mgcv* package [59]. For depth, we employed a Gaussian distribution, while for activity level and maximum daily displacement, we utilized a Tweedie error distribution. Full models included MHW stage, species, their interaction and body length as fixed effects. To account for individual behaviour and event-specific variability, individual fish identity and the specific MHW event were included as random effects. Models were progressively simplified to identify the most parsimonious fit via AIC (see supplementary material, table S3). Across all analyses, when candidate models were closely ranked (ΔAIC < 2), we selected the most parsimonious model to avoid overfitting and the inclusion of uninformative parameters [60].

#### (ii) Modelling diel patterns

Beyond comparing mean values, we explored potential diel changes in activity levels and depth (raw measurements) across MHW stages using generalized additive mixed models (GAMMs), which facilitate the analysis of complex nonlinear patterns. Depth measurements were log-transformed to satisfy GAMM assumptions, while activity was modelled using a Tweedie distribution. To ensure sufficient data density and reduce temporal bias, we applied the two or more detections per hour filter, excluding individual–MHW combinations with <100 detections per stage, and thinned data to one detection per hour per individual to reduce temporal autocorrelation. Visual inspections were used to exclude individuals lacking consistent coverage across the entire diel cycle. To account for seasonal differences in daylight, time of day was coded on a continuous scale anchored to four solar events (1 = nadir, 2 = sunrise, 3 = solar noon and 4 = sunset), linearly interpolated to align behaviour with solar cues rather than clock time [47].

For the two response variables (i.e. activity level and depth), we compared a set of candidate models to assess how MHWs modulate established diel patterns. Base models (model 0; see supplementary material, Table S4, for detailed models) included a species-specific cyclic smooth for time of day, body length as a continuous predictor and random effects for individual fish (factor-smooth interaction with time of day) [61]. We then incorporated MHW effects through three primary structures: (i) adding the MHW stage as a fixed effect to capture overall shifts in magnitude (model 1); (ii) adding a smooth interaction between time of day and MHW stage to capture shifts in diel pattern shape (model 2); and (iii) full models combined both fixed and smooth terms while testing for different levels of species-specific complexity. These ranged from shared community-level responses to full interactions between MHW stage and species (models 4–6). Cyclic cubic splines were assigned a basis dimension (*k*) of 24 for species-specific diel rhythms and 8 for MHW-stage interactions. Models were compared using AIC. Best-fitting models for each behavioural metric were checked for autocorrelation and, if needed, refitted with a first-order autoregressive correlation structure. All analyses were conducted using the *mgcv* package, and model diagnostics were validated using the *gam.check* and *acf* functions. Diel analyses included 45 fish-MHW combinations (24 individuals, 12 MHWs) for activity and 31 combinations (17 individuals, 11 MHWs) for depth.

#### (iii) Linking behavioural shift to marine heatwave characteristics

We were also interested in examining whether behavioural responses are associated with MHW characteristics. To synthesize these characteristics into a single metric of MHW severity, we conducted a PCA and extracted the first principal component (PC1, explaining 64.3% of the variance). Then, we used linear models to examine the relationship between MHW-driven shifts in depth, activity level and maximum daily displacement against both the individual MHW characteristics and the composite MHW severity index (PC1). Behavioural shifts were quantified by comparing each individual's mean values during any specific MHW to its ‘before’ period to quantify the direct behavioural deviation from prior baseline conditions. Activity and daily displacement shifts were calculated as the log response-ratio (log RR = log(‘MHW’/‘before’)), where negative values indicate a decrease relative to the baseline. For depth, we calculated the difference in mean depth (‘MHW’ − ‘before’), where positive values reflect a shift to deeper waters, while negative values indicate a shift to shallower depths.

## 3. Results

**Figure 2.**
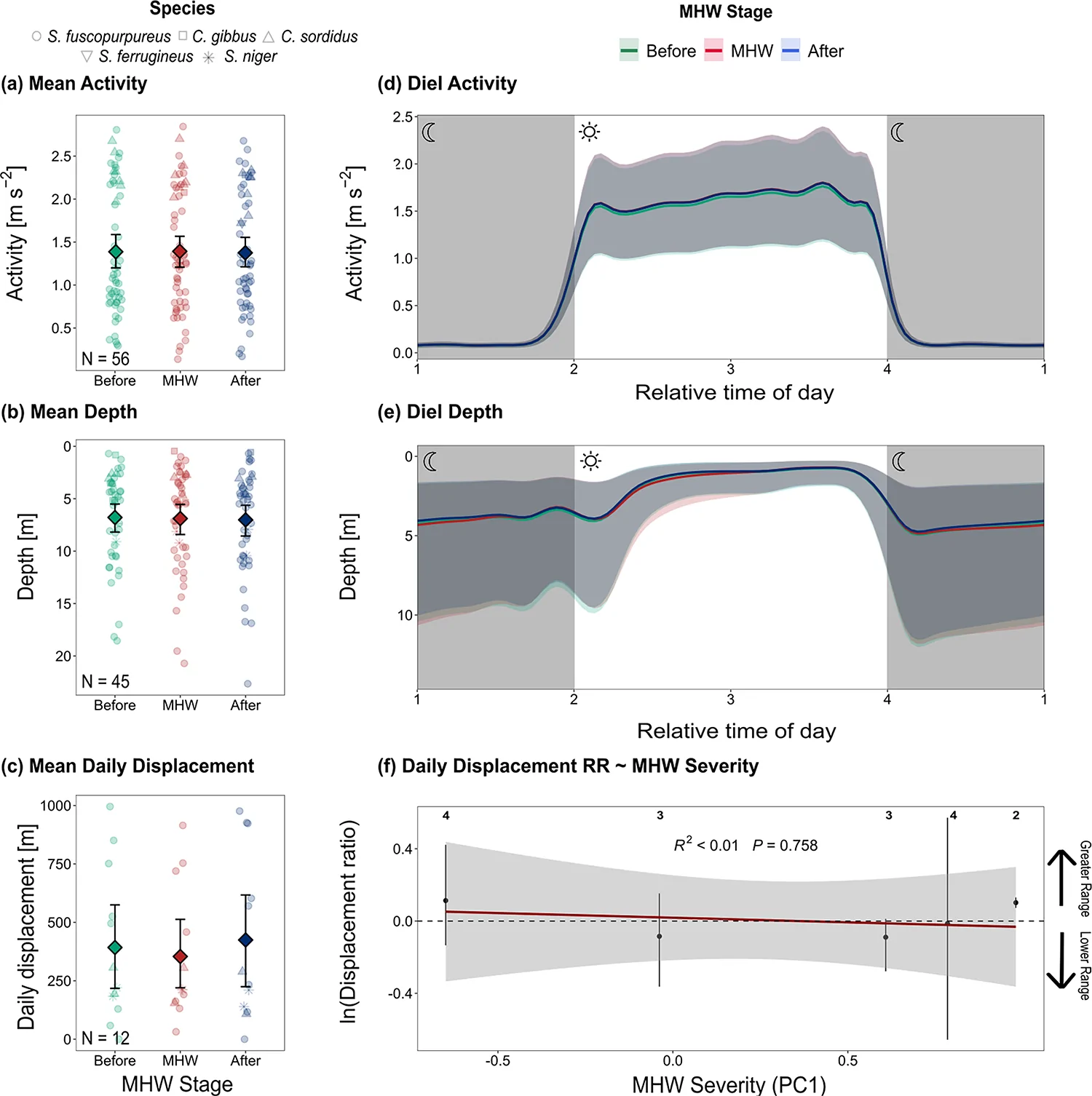
Behavioural responses of parrotfish to MHWs. (a) Activity, (b) depth and (c) daily displacement, before, during (denoted as MHW) and after MHWs. Small semi-transparent points denote individuals, while large solid rhombuses denote means. *N* refers to the number of MHW-individual combinations included in each analysis. (d) Activity and (e) depth predicted diel patterns for different MHW stages are plotted versus time of day (predicted for the most common species: *S. fuscopurpureus*). Values of the *x*-axis represent daily solar events (1 = nadir, 2 = sunrise, 3 = solar noon and 4 = sunset). Nighttime hours are shaded in grey for visual purposes. (f) Daily displacement log response-ratio (‘MHW’/’before’) as a function of MHW severity (estimated using PC1 of MHW characteristics). Points denote mean change for each MHW event, and the numbers on top represent the number of individuals in each event. Note that only five MHW events are presented in panel (f), as MHW no. 1 was excluded owing to the lack of baseline (‘before’) data required to calculate the response ratio. Throughout all panels, shaded ribbons represent 95% pointwise confidence bands, and error bars represent 95% confidence intervals. The colours green, red and blue represent the ‘before’, ‘during (MHW)’ and ‘after’ stages, respectively.

No significant differences were found in mean activity (figure 2a), depth (figure 2b) or daily displacement (figure 2c) across any of the three temporal stages (supplementary material, table S5).

The diel analysis revealed a significant nonlinear effect of time of day on both activity level and depth with distinct diel patterns for all species (*p* < 0.001). For activity, the best-fitting model included MHW stage solely as a fixed effect (model 1; 80.1% of the deviance explained; ΔAIC = 2.2 compared to the next best model, which also included a smooth term). However, this inclusion increased explanatory power by only 0.004% over the base model excluding the MHW stage. Activity levels were not influenced by MHW stage (ANOVA, *p* = 0.36) and fish length (ANOVA, *p* = 0.14) but varied significantly among species(ANOVA, *p* < 0.001). For depth, the top model incorporated the MHW stage both as a fixed effect and a smooth interaction with time of day (model 5, 75.1% of the deviance explained; ΔAIC = 5.2 compared to the next best model, which contained only a smooth term without the fixed effect), marginally increasing explained deviance by 0.06%. Consistent with the activity results, depth was not influenced by MHW stage (ANOVA, *p* = 0.09) or fish body length (ANOVA, *p* = 0.74) but was only influenced by species-specific differences (ANOVA, *p* = 0.036). Detailed model results are found in table S6, and individual model predictions are shown in figures S5 and S6 in the supplementary material.

No relationship was found between the change in activity level, depth and displacement and any of the MHW characteristics (figure 2f; see supplementary material, figure S3, for full results). Results for the detrended approach are provided in tables S7 and S8 and figures S7–S11 in the supplementary material. For isolated individual behavioural anomalies, see supplementary material.

## 4. Discussion

In this study, we examined parrotfishes' behavioural responses to MHWs in the northern Red Sea using long-term acoustic telemetry. To our knowledge, this is the first time a long-term acoustic dataset has been utilized to investigate the effects of extreme thermal events on *in situ* fish behaviour. We focused on three behavioural indices: depth utilization, activity level and daily displacement, comparing values before, during and after MHW events. Contrary to expectations, we found no significant behavioural changes; MHWs explained <1% of the behavioural variance, and the amount of change was independent of MHW severity. The lack of behavioural change associated with MHWs seems surprising given that elevated temperatures are known to alter metabolic rates in ectothermic organisms [62], with likely downstream effects on behaviour [63,64]. However, our results align with recent laboratory experiments showing little to no shifts in foraging behaviour of herbivorous fish during simulated MHWs [23,34].

Several factors may account for the lack of behavioural changes. Observed MHW events were primarily classified as moderate [65]. Even the warmest summer events reached a maximum absolute intensity of only 29.4°C. Notably, all recorded temperatures remained within the known preferred temperature ranges of the studied species (based on large-scale geographic distribution [66]). A rapid, lethal warming event in 2017 led to mass mortality of parrotfishes (including some of the specific species tracked in our study), which researchers linked to a disease outbreak caused by temperature-mediated immunosuppression [5]. Although this event occurred within our study timeframe, no tracking data were available from that exact period for behavioural analysis. The event itself did not meet the MHW criteria (maximum intensity of 28.6°C), yet was characterized by an extreme warming rate of 1.68°C day⁻¹ (a rate of temperature increase much steeper than the onset rates of the detected MHWs). The high mortality observed during this extreme event indicates that these parrotfishes did not successfully relocate to cooler refugia. This susceptibility suggests that the species lacked the selective pressure required to evolve behavioural avoidance in response to rapid warming. Furthermore, while parrotfishes may be physically capable of reaching deeper thermal refugia when available (such as below the approx. 40 m summer mixed-layer [67]; maximum tracking depth 55 m), utilizing them would require these obligate reef-dwellers to abandon their established shallow-water home-range [47]. Potential costs, such as reduced benthic algal availability at depth and the loss of prime grazing habitat, may outweigh the thermal benefits during moderate MHWs. Consequently, during moderate MHWs, parrotfish may rely on internal physiological or molecular responses [24,26,68] to buffer the effects of MHWs, averting the need for behavioural modifications.

The lack of pronounced behavioural shifts associated with MHWs highlights the complexity of thermal stress responses. We initially hypothesized that behavioural changes would serve as a warning sign before severe consequences such as mortality occur [4,5,69]. However, our results suggest that species vulnerability to MHWs may not be reliably predicted by behaviour alone, since a lack of behavioural response is inherently ambiguous and could reflect either true thermal resilience or extreme vulnerability. While these conclusions must be interpreted cautiously owing to analytical constraints (e.g. species heterogeneity and limited sample sizes), our findings concur with recent laboratory studies. Such studies detected minimal behavioural responses during simulated MHWs, yet reported loss of body mass driven by increased metabolic demands [23,34]. If this pattern of behavioural rigidity alongside physiological stress and reduced fitness is corroborated by field studies, it may signal hidden vulnerabilities in natural populations.

## Supplementary Material



## Data Availability

All acoustic telemetry datasets, environmental data (Sea Surface Temperature records) and R scripts required to reproduce the analyses and figures presented in this study are openly available in the Open Science Framework (OSF) repository [70]. Supplementary material is available online [71].
