# Supplementary material for: Parrotfish demonstrate little behavioural shift during marine heatwaves
Source: Biol Lett. 2026 Sep 9;22(9):20260337. doi: 10.1098/rsbl.2026.0337 (PMC13561080; doi:10.1098/rsbl.2026.0337)
Supplement: Supplementary file 1 [file rsbl.2026.0337.zip › rsbl.2026.0337/rsbl20260337_si_001.docx]

# Parrotfish demonstrate little behavioral shift during marine heatwaves - Supplementary material extended results

Dan B. Golanski^1,2^, Renanel Pickholtz^1,3^, Moshe Kiflawi^3,4^, Glenn T. Crossin^5^, Gitai Yahel^2^ and Jonathan Belmaker^1,6^

^1^ School of Zoology, George S. Wise Faculty of Life Sciences, Tel-Aviv University, Tel Aviv 6997801, Israel

^2^ Faculty of Marine Sciences, Ruppin Academic Center, Mikhmoret 4029700, Israel

^3^ The Interuniversity Institute for Marine Sciences of Eilat, 88103, Eilat, Israel

^4^ Department of Life-Sciences, Ben-Gurion University of the Negev, POB 653, 84105, Beer-Sheva, Israel

^5^ Department of Biology, Dalhousie University, 1355 Oxford Street, Halifax, Nova Scotia, B4H 4R2 Canada

^6^ Steinhardt Museum of Natural History, Tel-Aviv University, Tel Aviv 6997801, Israel

**Table S1: List of MHWs and their characteristics based on the fixed baseline definition.** Dates are shown in mm/dd/yyyy format**.** The 'MHW Start date' and 'MHW End date' refer exclusively to the actual thermal event (the 'during' phase) and do not include the associated 'before' or 'after' tracking periods. The Abbreviations: *SFu* = *Scarus fuscopurpureus*; *SFe = Scarus ferrugineus*; *SN = Scarus niger; CG* = *Chlorurus gibbus*; *CS = Chlorurus sordidus.*

| **#** | **MHW Start date** | **MHW End date** | **Duration (days)** | **Mean intensity (°C)** | **Maximum intensity (°C)** | **Cumulative intensity (°C·days)** | **Maximum absolute intensity (°C)** | **Onset rate (°C day⁻¹)** | **Category** | **Total fish N** | ***SFu N*** | ***CG N*** | ***SFe N*** | ***CS N*** | ***SN N*** |
| --- | --- | --- | --- | --- | --- | --- | --- | --- | --- | --- | --- | --- | --- | --- | --- |
| I | 07/04/2016 | 07/25/2016 | 22 | 1.5 | 1.9 | 33.2 | 28.5 | 0.08 | II Strong | 2 | - | - | 1 | 1 | - |
| II | 08/01/2016 | 08/23/2016 | 23 | 1.3 | 1.5 | 29.6 | 28.8 | 0.03 | I Moderate | 5 | - | 1 | 1 | 1 | 2 |
| III | 04/06/2017 | 04/12/2017 | 7 | 0.9 | 1.1 | 6.5 | 22.6 | 0.17 | I Moderate | 7 | 6 | - | - | 1 | - |
| IV | 02/03/2018 | 02/11/2018 | 9 | 1.0 | 1.2 | 9.3 | 22.6 | 0.11 | I Moderate | 8 | 6 | - | - | 2 | - |
| V | 02/20/2018 | 02/27/2018 | 8 | 0.9 | 0.9 | 6.8 | 22.1 | 0.03 | I Moderate | 8 | 6 | - | - | 2 | - |
| VI | 03/04/2018 | 03/27/2018 | 24 | 1.1 | 1.3 | 26.0 | 22.6 | 0.08 | I Moderate | 6 | 5 | - | - | 1 | - |
| VII | 06/25/2019 | 07/19/2019 | 25 | 1.4 | 1.7 | 34.7 | 28.0 | 0.07 | I Moderate | 1 | - | - | - | 1 | - |
| VIII | 08/05/2019 | 09/02/2019 | 29 | 1.6 | 2.1 | 48.0 | 29.4 | 0.09 | I Moderate | 3 | 3 | - | - | - | - |
| IX | 09/06/2019 | 09/24/2019 | 19 | 1.3 | 1.5 | 24.5 | 28.1 | 0.03 | I Moderate | 3 | 2 | 1 | - | - | - |
| X | 01/02/2020 | 01/24/2020 | 23 | 0.9 | 1.1 | 21.9 | 23.2 | 0.02 | I Moderate | 8 | 8 | - | - | - | - |
| XI | 02/14/2020 | 02/18/2020 | 5 | 0.8 | 0.8 | 3.9 | 22.0 | 0.03 | I Moderate | 10 | 8 | 2 | - | - | - |
| XII | 11/23/2020 | 11/28/2020 | 6 | 0.9 | 1.0 | 5.7 | 24.8 | 0.05 | I Moderate | 3 | 3 | - | - | - | - |

**Table S2: Tagged Fish metadata** **and analyses inclusion**. Abbreviations: *SFu* = *Scarus fuscopurpureus*; *SFe = Scarus ferrugineus; SN = Scarus niger*; *CG* = *Chlorurus gibbus*; *CS = Chlorurus sordidus.* Asterisks denote missing data, and hashtags denote fish that were also included in the detrended baseline analysis.

| **Fish id** | **Species** | **Fork Length (cm)** | **Weight (g)** | **Number of MHWs**  **(Fix)** | **Mean depth analysis** | **Mean activity analysis** | **Mean displacement analysis** | **Diel activity analysis** | **Diel depth analysis** |
| --- | --- | --- | --- | --- | --- | --- | --- | --- | --- |
| 19111339^#^ | *SFu* | 35.5 | 900 | 2 | V | V | - | V | V |
| 19111342 | *SFu* | 36.5 | 818 | 3 | V | V | - | V | V |
| 19111348 | *SFu* | 35.3 | 860 | 3 | V | V | - | V | V |
| 19111351 | *SFu* | 35.3 | 850 | 3 | V | V | - | V | V |
| 19111354 | *SFu* | 36.4 | 813 | 2 | V | V | - | V | V |
| 19111357^#^ | *SFu* | 34.2 | 858 | 4 | V | V | - | V | V |
| 19111363 | *SFu* | 36.2 | 880 | 2 | V | V | - | V | V |
| 19111369 | SFu | 34.0 | 685 | 2 | V | V | - | V | V |
| 19111372 | SFu | 37.1 | 900 | 2 | V | V | - | V | V |
| 1255785 | *SFu* | 36.4 | 966 | 1 | V | V | V | - | - |
| 1255791 | *SFu* | * | * | 1 | - | - | - | V | - |
| 1255800^#^ | SFu | 37.0 | 1050 | 4 | V | V | V | V | - |
| 1255801 | SFu | 36.4 | 840 | 1 | V | V | V | V | V |
| 1255802 | SFu | 40.5 | 1180 | 1 | V | V | V | V | V |
| 1255803 | SFu | 37.2 | 1150 | 1 | V | V | V | - | - |
| 1255804 | SFu | 38.8 | 1085 | 1 | V | V | - | - | V |
| 1255806^#^ | *SFu* | 38.6 | 1104 | 4 | V | V | V | V | V |
| 1255807 | *SFu* | 38.4 | 1050 | 3 | - | V | - | V | - |
| 1255810^#^ | *SFu* | 37.9 | 940 | 3 | V | V | - | V | - |
| 1255811^#^ | *SFu* | 36.8 | 854 | 3 | V | V | - | V | - |
| 1255812 | *SFu* | 39.5 | 870 | 2 | V | V | - | V | V |
| 1212925^#^ | *SFe* | 36.0 | * | 2 | - | V | - | V | - |
| 1168792 | *SN* | 30.3 | * | 2 | V | - | V | - | V |
| 1168793 | *SN* | 34.5 | * | 1 | V | - | V | - | - |
| 1212923 | *CG* | 29.7 | * | 1 | - | V | - | - | - |
| 1273484 | *CG* | 31.8 | 470 | 1 | V | - | - | - | - |
| 1273489 | *CG* | 34.3 | 830 | 2 | V | - | - | V | V |
| 1255814^#^ | *CS* | 52.0 | >1500 | 2 | - | V | - | V | - |
| 1255815^#^ | *CS* | 49.4 | >1500 | 3 | V | V | V | V | - |
| 1212924^#^ | *CS* | 28.5 | * | 2 | - | V | V | V | - |
| 1255784 | *CS* | 34.0 | 605 | 1 | V | V | - | - | - |
| 1255792^#^ | *CS* | 30.0 | 440 | 1 | V | V | - | V | - |

**Figure S1.** **SST and fixed baseline MHWs time series at Eilat from 2016 to 2020.** The lines indicate surface seawater temperature (black line), its seasonal climatology (blue dashed line), and marine heatwave threshold (red dot-dashed line). Periods of MHWs are highlighted with a semi-transparent red band, and the area between the observed temperature and the threshold during MHWs is filled in red. Note that several periods exceeding the temperature threshold are not highlighted as MHWs because they did not meet the duration criteria (i.e., events lasting less than 5 days or exceeding 1 month). The numbers above each MHW fit to the numbers in Table S1.

**Table S3: List of GLMMs used to examine the change in mean behavioral metrics across MHWs stages (Fix baseline).** AIC values were calculated for the 1-week ‘before’ and ‘after’ timeframes. Chosen models are shown in bold. Note that even though some models have a lower AIC value, when candidate models were closely ranked (ΔAIC<2), we selected the most parsimonious model to avoid overfitting and the inclusion of uninformative parameters.

| **Behavioral Metric** | **Model Step** | **Model Formula** | **AIC** | **ΔAIC** |
| --- | --- | --- | --- | --- |
| Activity | Full model | mean_activity ~ Before_After * species + scale(Length_cm) + s(fish_id, bs = "re") + s(Serial, bs = "re") | -22.99 | 0 |
|  | Main effects model | mean_activity ~ Before_After + species + scale(Length_cm) + s(fish_id, bs = "re") + s(Serial, bs = "re") | -22.39 | 0.59 |
|  | **Parsimonious model** | mean_activity ~ Before_After + species + s(fish_id, bs = "re") + s(Serial, bs = "re") | -22.52 | 0.46 |
| Depth | Full model | mean_depth ~ Before_After * species + scale(Length_cm) + s(fish_id, bs = "re") + s(Serial, bs = "re") | 436.35 | 10.70 |
|  | Main effects model | mean_depth ~ Before_After + species + scale(Length_cm) + s(fish_id, bs = "re") + s(Serial, bs = "re") | 425.66 | 0 |
|  | **Parsimonious model** | mean_depth ~ Before_After + species + s(fish_id, bs = "re") + s(Serial, bs = "re") | 425.66 | 0 |
| Maximum Displacement | Full model | mean_disp_max ~ Before_After * species + scale(Length_cm) + s(fish_id, bs = "re") + s(Serial, bs = "re") | 421.60 | 12.09 |
|  | Main effects model | mean_disp_max ~ Before_After + species + scale(Length_cm) + s(fish_id, bs = "re") + s(Serial, bs = "re") | 409.5 | 0 |
|  | **Parsimonious model** | mean_disp_max ~ Before_After + species + s(fish_id, bs = "re") + s(Serial, bs = "re") | 409.65 | 0.14 |

**Table S4: List of GAMMs used to examine the shifts in diel patterns associated with MHWs.** The response variable (y) represents either log-transformed activity or depth. All models include fish length and a random intercept for MHW events (Serial). Diel patterns are modeled using cyclic cubic splines for time of day. Individual variability is accounted for using a factor-smooth interaction for each fish ID.

| # | **Model name** | **Model formula** | **Model description** |
| --- | --- | --- | --- |
| 0 | Base | y ~ scale(length) + species + s(Time of day, by = species, bs='cc') + s(fish id, bs ='fs") | **Baseline**: Behavior follows species-specific diel cycles and individual variation, with no MHW effect. |
| 1 | MHW Intercept | Base + MHW Stage | **Shift in Magnitude** MHW causes a uniform shift (increase/decrease) in the mean behavioral level across all species |
| 2 | MHW Intercept X Species | Base + MHW Stage * species | **Species-specific Shift:** MHW causes a shift in the mean behavioral level, but the direction or magnitude differs by species. |
| 3 | MHW Smooth | Base + s(Time of day, by = MHW stage, bs = ‘cc’) | **Shift in Diel Shape:** MHW changes the shape of the daily rhythm (e.g., shift in peak activity hours) regardless of species |
| 4 | Full (No species) | y ~ scale(length) + s(fish id, bs ='fs") +MHW Stage + s(Time of day, by = MHW stage, bs = ‘cc’) | **Combined Shift:** MHW affects both the mean behavioral level and the shape of the diel cycle uniformly for all species. |
| 5 | Full + Species | Base + MHW Stage + s(Time of day, by = MHW stage, bs = ‘cc’) | **Species-Specific Rhythm:** MHW shifts both mean and rhythm, while accounting for baseline species differences. |
| 6 | Full Interaction | y ~ scale(length) + species * MHW Stage + s(Time of day, by = species, bs='cc') + s(fish id, bs ='fs") + s(Time of day, by = MHW stage, bs = ‘cc’) | **Comprehensive Response:** The most complex model; MHW affects mean levels and diel shapes, with the mean shift being species-specific. |

**Table S5: Summary of the overall mean parsimonious models (GLMMs) evaluating the effect of MHWs on three parrotfish behavioral metrics.** Models display the results of the 1-week duration window analysis. Baseline reference levels are 'before' for MHW stage, and *S. fuscopurpureus* for species. Models were fitted using the *mgcv* package. The response variables were modeled with a log link function. Variance components and standard deviations (SD) with 95% confidence intervals (CI) were extracted for the random effects (fish_id and MHW Serial) using the gam.vcomp function. For the Maximum Displacement model, the variance associated with MHW Serial approached zero, resulting in a singular fit; thus, confidence intervals for this specific random term are not applicable (N/A). Significant terms are colored red, and values are rounded up to two decimal digits.

| **Variable** | **Component** | **Term** | **Estimate** | **Std. error** | **t-value** | **p-value** |
| --- | --- | --- | --- | --- | --- | --- |
| **Activity**  Link function: log | Parametric effects | Intercept (Baseline: *S. fuscopurpureus*, Before) | 0.07 | 0.13 | 0.58 | 0.56 |
|  |  | Stage [MHW] | 0.004 | 0.03 | 0.13 | 0.90 |
|  |  | Stage [After] | -0.005 | 0.03 | -0.15 | 0.88 |
|  |  | Species [*C. gibbus*] | 0.74 | 0.55 | 1.33 | 0.18 |
|  |  | Species [*C. sordidus*] | 0.64 | 0.29 | 2.21 | 0.03 |
|  |  | Species [*S. ferrugineus*] | 0.17 | 0.55 | 0.30 | 0.76 |
|  | Random effects |  | **Variance** | **SD** | **CI (SD) 95%** | **N** |
|  |  | Fish id | 0.27 | 0.52 | [0.37,0.72] | 22 |
|  |  | MHW Serial | 0.02 | 0.14 | [0.05,0.39] | 10 |
|  |  | Residuals | 0.03 | 0.18 | [0.16,0.20] | 168 |
| **Depth**  Link function: log |  | **Term** | **Estimate** | **Std. error** | **t-value** | **p-value** |
|  | Parametric effects | Intercept (Baseline: *S. fuscopurpureus*, Before) | 1.69 | 0.18 | 9.55 | <0.001 |
|  |  | Stage [MHW] | 0.04 | 0.03 | 1.51 | 0.13 |
|  |  | Stage [After] | 0.05 | 0.03 | 1.80 | 0.07 |
|  |  | Species [*C. gibbus*] | -0.84 | 0.59 | -1.42 | 0.16 |
|  |  | Species [*C. sordidus*] | -0.51 | 0.54 | -0.94 | 0.35 |
|  |  | Species [*S. niger*] | 0.44 | 0.56 | 0.80 | 0.44 |
|  | Random effects |  | **Variance** | **SD** | **CI (SD) 95%** | **N** |
|  |  | Fish id | 0.50 | 0.71 | [0.51,0.99] | 22 |
|  |  | MHW Serial | 0.03 | 0.16 | [0.08,0.31] | 8 |
|  |  | Residuals | 1.08 | 1.04 | [0.91,1.19] | 135 |
| **Maximum**  **Displacement**  Link function: log |  | **Term** | **Estimate** | **Std. error** | **t-value** | **p-value** |
|  | Parametric effects | Intercept (Baseline: *S. fuscopurpureus*, Before) | 5.32 | 0.51 | 10.40 | <0.001 |
|  |  | Stage [MHW] | -0.10 | 0.09 | -1.09 | 0.28 |
|  |  | Stage [After] | 0.08 | 0.09 | 0.87 | 0.39 |
|  |  | Species [*C. sordidus*] | 0.05 | 1.00 | 0.05 | 0.96 |
|  |  | Species [*S. niger*] | -0.04 | 1.00 | -0.04 | 0.97 |
|  |  |  | **Variance** | **SD** | **CI (SD) 95%** | **N** |
|  | Random effects | Fish id | 1.49 | 1.22 | [0.67,2.22] | 8 |
|  |  | MHW Serial | ~0.00 | ~0.00 | NA | 5 |
|  |  | Residuals | 17.92 | 4.23 | [4.15,4.32] | 36 |

**Table S6: Summary of the diel behavior models(GAMMs).** Models display the results of the 1-week duration window analysis. Baseline reference levels are 'Before' for Stage, and *S. fuscopurpureus* for Species. Models were fitted using the *mgcv* package. Activity was modeled using a Tweedie distribution with a log link function, while Depth was log-transformed (x + 0.05) before analysis. The table presents parametric coefficients for fixed effects alongside the approximate significance for smooth terms. Inter-individual variation and inter-MHW variation were accounted for by including a random smooth for fish identity (s(Time of Day, Fish ID)) and a random intercept for MHW sequence (s(MHW serial)), respectively. Significant terms are colored red, and values are rounded up to two decimal digits.

| **Variable** | **Component** | **Term** | **Estimate** | **Std. error** | **t-value** | **p-value** |
| --- | --- | --- | --- | --- | --- | --- |
| **Activity**  Link function: log  **(Model 1)** | Parametric effects | Intercept (Baseline: *S. fuscopurpureus*, Before) | -0.87 | 0.06 | -15.33 | <0.001 |
|  |  | Stage [MHW] | 0.02 | 0.02 | 1.39 | 0.16 |
|  |  | Stage [After] | 0.02 | 0.02 | 1.17 | 0.24 |
|  |  | Species [*C. sordidus*] | 0.53 | 0.13 | 4.15 | <0.001 |
|  |  | Species [*S. ferrugineus*] | 0.41 | 0.16 | 2.54 | 0.01 |
|  |  | Body Length (scaled) | 0.03 | 0.02 | 1.48 | 0.14 |
|  | Smooth terms | **Term** | **edf** |  | **F-value** | **p-value** |
|  |  | s(Time of Day) × *S. fuscopurpureus* | 21 |  | 86.17 | <0.001 |
|  |  | s(Time of Day) × *C. sordidus* | 16.78 |  | 8.59 | <0.001 |
|  |  | s(Time of Day) × *S. ferrugineus* | 15.5 |  | 5.63 | <0.001 |
|  |  | s(Time of Day, Fish ID) [Random smooth] | 129.2 |  | 11.35 | <0.001 |
|  |  | s(MHW serial) [Random intercept] | <0.001 |  | 0 | 0.509 |
|  | Parametric effects | **Term** | **Estimate** | **Std. error** | **t-value** | **p-value** |
| **Depth**  Log transformed  (x + 0.05)  **(Model 5)** |  | Intercept (Baseline: *S. fuscopurpureus*, Before) | 0.87 | 0.25 | 3.46 | <0.001 |
|  |  | Stage [MHW] | 0.00 | 0.02 | 0.18 | 0.85 |
|  |  | Stage [After] | -0.04 | 0.02 | -1.58 | 0.11 |
|  |  | Species [*C. sordidus*] | 0.59 | 0.89 | 0.66 | 0.51 |
|  |  | Species [*S. ferrugineus*] | -1.55 | 0.69 | -2.26 | 0.02 |
|  |  | Species [*S. niger*] | 0.98 | 0.89 | 1.11 | 0.27 |
|  |  | Body Length (scaled) | 0.04 | 0.12 | 0.32 | 0.74 |
|  | Smooth terms | **Term** | **edf** |  | **F-value** | **p-value** |
|  |  | s(Time of Day) × *S. fuscopurpureus* | 19.65 |  | 12.30 | <0.001 |
|  |  | s(Time of Day) × *C. sordidus* | 16.04 |  | 3.72 | <0.001 |
|  |  | s(Time of Day) × *S. ferrugineus* | 19.99 |  | 7.86 | <0.001 |
|  |  | s(Time of Day) × *S. niger* | 8.34 |  | 0.96 | <0.001 |
|  |  | s(Time of Day, Fish ID) [Random smooth] | 111.4 |  | 68.27 | <0.001 |
|  |  | s(Time of Day) × Stage [Before] | <0.001 |  | 0 | 0.85 |
|  |  | s(Time of Day) × Stage [After] | <0.001 |  | 0 | 0.80 |
|  |  | s(Time of Day) × Stage [MHW] | 4.782 |  | 2.92 | <0.01 |
|  |  | s(MHW serial) [Random intercept] | 0.916 |  | 10.89 | <0.001 |

### Fixed baseline - extended results

**Figure S2. Distribution of MHW characteristics included in this study under the fixed baseline definition**. Due to the differences in scales, the characteristics were divided into two panels: (a) large-scale descriptors and (b) small-scale descriptors. Boxes represent the interquartile range with medians (horizontal lines), whiskers extend to the nearest data point that is less than 1.5 IQR from the box limits, and points indicate individual events.

**Figure S3. Linear relationships between parrotfish behavioral responses and MHW characteristics**. Panels (a) activity, (b) depth, and (c) daily displacement, comparing behavior during MHW to the 'before' stage. Activity and daily displacement are expressed as the log ratio of the mean value during the MHW to the mean value before the event. Depth is expressed as the difference between mean depths in the two periods. Points represent mean responses across individual MHWs with error bars indicating 95% confidence intervals. Red lines show linear fits with shaded ribbons denoting 95% confidence intervals. For each model, the R² and p-value are shown in the top left corner. Note that this comparative analysis required valid tracking data from both the 'before' and 'MHW' stages. Due to missing data from the 'before' stage or from specific sensors for certain events, the number of MHWs analyzed varies across metrics: activity includes 10 events, depth includes 9 events, and daily displacement (which is based exclusively on the first acoustic array deployment period) includes 5 events.

**Figure S4. Behavioral responses of parrotfish to marine heatwaves - raw data (fixed baseline).** (a) Activity and (b) depth before, during, and after MHWs. Points represent raw observations, and solid lines denote GAMM-predicted diel patterns for each MHW stage. Values on the x-axis correspond to the anchored solar events of time of day (1 = nadir, 2 = sunrise, 3 = solar noon, 4 = sunset). The colors green, red, and blue represent the ‘before’, ‘during’ (MHW), and ‘after’ stages, respectively. The smooths are the GAMM predictions for the most common species: *S. fuscopurpureus.*

**Figure S5. Depth responses of individual fish to marine heatwaves (fixed baseline)**. GAMM predicted diel depth patterns for each fish across different MHW stages. Values of the x-axis represent daily solar events (1 = nadir, 2 = sunrise, 3 = solar noon, and 4 = sunset). Shaded gray areas indicate nighttime hours for reference. The colors green, red, and blue represent the ‘before’, ‘during’ (MHW), and ‘after’ stages, respectively. Numbers on top of each facet denote the MHW_serial_number_Fish ID. Note that the y-axis scale isn't uniform due to the unique depth distribution of each individual.

**Figure S6. Shift in individual daily activity patterns during and after marine heatwaves (fixed baseline).** To isolate and visualize the specific effect of MHWs on fish activity, predictions are presented as the difference (delta) from the pre-MHW baseline. Because the best-fit GAMM for activity indicates a uniform shift in magnitude without altering the overall shape of the diel cycle, plotting absolute activity values obscures these subtle shifts beneath the large amplitude of natural daily rhythms. Here, the horizontal dashed black line (delta = 0) represents the 'before' baseline. Colored solid lines denote the predicted deviation from this baseline 'during' the MHW (red) and 'after' the MHW (blue). Values on the x-axis represent daily solar events (1 = nadir, 2 = sunrise, 3 = solar noon, and 4 = sunset). Shaded gray areas indicate nighttime hours for reference. Numbers on top of each facet denote the MHW serial number and Fish ID Fish with no data during the ‘before’ period were excluded from the plot.

### Detrended baseline results

Using the detrended baseline approach, we identified four MHWs that matched the temporal requirements and had sufficient acoustic data to be included in the analysis. These events had a mean duration of 15 ± 7.2 days (mean ± 95% CI), with absolute maximum temperatures ranging from 25.5 °C to 29.4 °C. The highest recorded temperature anomaly (maximum intensity) was 2.2 °C, and the highest cumulative intensity reached 21.4 °C days (Table S7 and Figs. S7-8). Each analyzed MHW included between 2 and 8 tracked individuals.

Data limitations across all detrended baselines precluded the analysis of daily displacement. For overall mean analyses, the final sample sizes comprised 10 and 9 fish-MHW combinations for activity and depth, respectively. Using the fixed 1-week and 1.5-multiplier baseline windows, no significant differences were observed in mean activity (Fig. S9a) across MHW stages, with model selection favoring the parsimonious model (Table S7). However, extending the baseline to a 3-multiplier window yielded significant effects for activity. Model selection for this broader window identified the full model as the best fit, revealing a significant interaction between MHW stage and species (p = 0.049). Specifically, *C. sordidus* exhibited an apparent higher mean activity level in the post-MHW period (an increase of 0.7 m s⁻²). Further inspection revealed this effect to be an artifact of diel sampling bias rather than a true behavioral shift. One of the two *C. sordidus* individuals in this dataset (MHW Serial 4; fish ID 1255792) was detected almost exclusively during inactive night and twilight hours prior to and during the MHW. Following receiver maintenance in the extended post-MHW period, detections for this individual occurred predominantly during midday peak activity hours. The absence of naturally higher daytime activity values in the pre- and during-MHW periods artificially deflated those means, driving the apparent post-MHW increase and the resulting statistical significance of the interaction term.

For mean depth, model selection favored the parsimonious model across temporal windows, and a significant influence of MHW stage (p < 0.01) was observed. The highest difference in depth was a reduction of 1.5m during the MHW (1-week window; Parsimonious model; p < 0.01). Despite being statistically significant, the small effect size and the lack of biological importance of this shift in terms of thermal refugee render both the magnitude and direction of this change biologically negligible.

Due to a highly limited sample size for depth (only 5 individuals), the diel analysis under the detrended framework was conducted exclusively for activity level (comprising 9 individuals across 9 fish-MHW combinations. Model selection favored the model that incorporated the MHW stage only as a smooth interaction with time of day (Model 3; 79.7% of the deviance explained; ΔAIC = 15.3 compared to the next best model, which excludes the MHW stage term completely), meaning the model doesn't indicate a change in the mean activity level due to the different MHW stages. However, similar to the main analysis, the change in explanatory power associated with the MHW was marginal, with deviance explained increasing by only 0.05% compared to a baseline model excluding the MHW stage. While clear diel patterns were maintained across all MHW stages, the shape of these daily trajectories differed significantly among species (p < 0.001) and exhibited a distinct shift during the post-MHW period (p = 0.039; Fig. S9c). Finally, species identity and fish length significantly influenced baseline activity levels (ANOVA, p = 0.04 & p = 0.01, respectively). Individual model predictions are shown in Fig. S11.

Finally, due to the smaller sample size of marine heatwaves and individuals evaluated under the detrended baseline, we could not robustly test for linear relationships between behavioral responses and MHW characteristics within this framework.

**Table S7: List of MHWs and their characteristics based on the detrended baseline definition.** Dates are shown in mm/dd/yyyy format. The 'MHW Start date' and 'MHW End date' refer exclusively to the actual thermal event (the 'during' phase) and do not include the associated 'before' or 'after' tracking periods. Abbreviations: *SFu* = *Scarus fuscopurpureus*; *CG* = *Chlorurus gibbus*; *SFe = Scarus ferrugineus*; *CS = Chlorurus sordidus; SN = Scarus niger.*

| **#** | **Start date** | **End date** | **Duration (days)** | **Mean intensity (°C)** | **Maximum intensity (°C)** | **Cumulative intensity (°C·days)** | **Maximum absolute intensity (°C)** | **Onset rate**  **(°C day⁻¹)** | **Fish N** | **SFu N** | **CG N** | **SFe N** | **CS N** | **SN N** |
| --- | --- | --- | --- | --- | --- | --- | --- | --- | --- | --- | --- | --- | --- | --- |
| I | 07/07/2016 | 07/26/2016 | 20 | 1.2 | 1.5 | 18.4 | 28.5 | 0.14 | 2 | - | - | 1 | 1 | - |
| II | 04/28/2018 | 05/12/2018 | 15 | 1.6 | 2.2 | 21.4 | 25.5 | 0.24 | 6 | 4 | - | - | 2 | - |
| III | 06/30/2019 | 07/08/2019 | 9 | 1.0 | 1.1 | 7.3 | 27.7 | 0.16 | 2 | 1 | - | 1 | - | - |
| IV | 08/06/2019 | 08/21/2019 | 16 | 1.2 | 1.5 | 12.3 | 29.4 | 0.21 | 4 | 3 | - | - | 1 | - |

**Figure S7*.*** ***SST and detrended baseline MHWs time series at Eilat from 2016 to 2020.*** The lines indicate surface seawater temperature (black line), its seasonal climatology (blue dashed line), and marine heatwave threshold (red dot-dashed line). Periods of MHWs are highlighted with a semi-transparent red band, and the area between the observed temperature and the threshold during MHWs is filled in red. Note that several periods exceeding the temperature threshold are not highlighted as MHWs because they did not meet the duration criteria (i.e., events lasting less than 5 days or exceeding 1 month). The numbers above each MHW fit to the numbers in table S7.

**Figure S8. Distribution of MHW characteristics included in this study under the detrended baseline definition**. Due to the differences in scales, the characteristics were divided into two panels: (a) large-scale descriptors and (b) small-scale descriptors. Boxes represent the interquartile range with medians (horizontal lines), whiskers extend to the nearest data point that is less than 1.5 IQR from the box limits, and points indicate individual events.

**Table S8: List of GLMMs used to examine the change in mean behavioral metrics across MHWs stages (Detrended baseline).** AIC values were calculated for the 1 week ‘before’ and ‘after’ timeframes. Chosen models are shown in bold. Note that even though some models have a lower AIC value, when candidate models were closely ranked (ΔAIC<2), we selected the most parsimonious model to avoid overfitting and the inclusion of uninformative parameters.

| **Behavioral Metric** | **Model Step** | **Model Formula** | **AIC** | **ΔAIC** |
| --- | --- | --- | --- | --- |
| Activity | Full model | mean_activity ~ Before_After * species + scale(Length_cm) + s(fish_id, bs = "re") + s(Serial, bs = "re") | 4.41 | 0.17 |
|  | Main effects model | mean_activity ~ Before_After + species + scale(Length_cm) + s(fish_id, bs = "re") + s(Serial, bs = "re") | 4.24 | 0 |
|  | **Parsimonious model** | mean_activity ~ Before_After + species + s(fish_id, bs = "re") + s(Serial, bs = "re") | 4.29 | 0.04 |
| Depth | Full model | log(mean_depth) ~ Before_After * species + scale(Length_cm) + s(fish_id, bs = "re") + s(Serial, bs = "re") | 106.6 | 0 |
|  | Main effects model | log(mean_depth )~ Before_After + species + scale(Length_cm) + s(fish_id, bs = "re") + s(Serial, bs = "re") | 106.86 | 0.26 |
|  | **Parsimonious model** | log(mean_depth )~ Before_After + species + s(fish_id, bs = "re") + s(Serial, bs = "re") | 106.87 | 0.27 |

**Figure S9. Behavioral responses of parrotfish to marine heatwaves under the detrended baseline**. (a) Depth and (b) Activity, before, during (denoted as MHW), and after marine heatwaves. Small semi-transparent points denote individuals, while large solid rhombuses denote means. N refers to the number of individuals included in each analysis. (c) Activity predicted diel patterns for different MHW stages are plotted versus time of day (predicted for the most common species: *S. fuscopurpureus*). Values of the x-axis represent daily solar events (1 = nadir, 2 = sunrise, 3 = solar noon, and 4 = sunset). Nighttime hours are shaded in gray for visual purposes. Throughout all panels, error bars represent 95% confidence intervals. The colors green, red, and blue represent the ‘before’, ‘during’ (MHW), and ‘after’ stages, respectively. Note that the 'before' curve isn't visible due to the lack of change between the 'before' and the MHW periods.

**Figure S10. Diel activity patterns of parrotfish to marine heatwaves - raw data (detrended baseline).** Activity levels before, during, and after MHWs. Points represent raw observations, and solid lines denote GAMM-predicted diel patterns for each MHW stage. Values on the x-axis correspond to the anchored solar events of time of day (1 = nadir , 2 = sunrise, 3 = solar noon, 4 = sunset). The colors green, red, and blue represent the ‘before’, ‘during’ (MHW), and ‘after’ stages, respectively. The smooths are the GAMM predictions for the most common species: *S*. *fuscopurpureus.* Note that the 'before' curve isn't visible due to the lack of change between the 'before' and the MHW periods.

**Figure S11. Individuals' activity responses to marine heatwaves (detrended baseline).** GAMM predicted diel activity patterns for each fish across different MHWs stages. Values of the x-axis represent daily solar events (1 = nadir, 2 = sunrise, 3 = solar noon, and 4 = sunset). Shaded gray areas indicate nighttime hours for reference. The colors green, red, and blue represent the ‘before’, ‘during’ (MHW), and ‘after’ stages, respectively. Numbers on top of each facet denote MHW serial number_Fish id. Note that the 'before' curve isn't visible due to the lack of change between the 'before' and the MHW periods.
