# Supplementary material for: Parrotfish demonstrate little behavioural shift during marine heatwaves
Source: Biol Lett. 2026 Sep 9;22(9):20260337. doi: 10.1098/rsbl.2026.0337 (PMC13561080; doi:10.1098/rsbl.2026.0337)
Supplement: Supplementary file 1 [file rsbl.2026.0337.zip › rsbl.2026.0337/rsbl20260337_si_002.docx]

# Parrotfish demonstrate little behavioral shift during marine heatwaves - Supplementary material sensitivity analyses & individual behavioral anomalies

Dan B. Golanski^1,2^, Renanel Pickholtz^1,3^, Moshe Kiflawi^3,4^, Glenn T. Crossin^5^, Gitai Yahel^2^ and Jonathan Belmaker^1,6^

^1^ School of Zoology, George S. Wise Faculty of Life Sciences, Tel-Aviv University, Tel Aviv 6997801, Israel

^2^ Faculty of Marine Sciences, Ruppin Academic Center, Mikhmoret 4029700, Israel

^3^ The Interuniversity Institute for Marine Sciences of Eilat, 88103, Eilat, Israel

^4^ Department of Life-Sciences, Ben-Gurion University of the Negev, POB 653, 84105, Beer-Sheva, Israel

^5^ Department of Biology, Dalhousie University, 1355 Oxford Street, Halifax, Nova Scotia, B4H 4R2 Canada

^6^ Steinhardt Museum of Natural History, Tel-Aviv University, Tel Aviv 6997801, Israel

### Sensitivity analyses

In addition to the standard 1-week fixed duration window presented in the main text, we conducted sensitivity analyses using a biologically scaled approach, defining the tracking windows as 1.5/3 times the duration of the respective MHW.

The rationale for testing both fixed and proportional windows lies in the complex behavioral and physiological trade-offs wild fishes face during thermal extremes. For organisms that remain within their shallow baseline habitats and modify only their activity levels, the dynamic balance between thermal damage accumulation and physiological repair rates means that longer exposure inevitably builds a higher thermal debt, requiring a proportionally longer recovery period to clear [1,2]. Prolonged exposure to extreme heat can create a severe energy deficit and deplete internal energy reserves in marine ectotherms [3]. Conversely, if fishes utilize behavioral thermoregulation by shifting to deeper, cooler waters, they could theoretically evade acute thermal damage altogether and resume baseline behaviors immediately following the event, which would favor a shorter, fixed observation window. However, such depth shifts may not be costless or entirely protective. Deepening can impose significant non-thermal fitness costs, such as reduced access to optimal foraging grounds or altered prey availability, which may accumulate an energetic deficit over time. Furthermore, deeper waters may still experience elevated temperatures during severe MHWs, providing only partial mitigation and slowing, rather than completely preventing, thermal debt accumulation. Because the exact interplay between behavioral mitigation, energetic costs, and physiological repair rates remains unknown for wild teleosts, applying a fixed 1-week window uniformly across all events may underrepresent the necessary recovery period for multi-week MHWs. Therefore, scaling the tracking windows proportionally to the MHW duration provides a robust framework to capture true carryover effects based on the accumulated thermal debt.

Our models demonstrated that the overarching behavioral patterns remained consistent regardless of whether the fixed 1-week, the proportional windows, or an exclusive subset of strictly isolated MHWs were applied (see Table S9). Importantly, while some of these alternative sensitivity models occasionally yielded statistically significant differences due to high data density, the actual effect sizes, representing the absolute magnitude of the behavioral shifts, were negligible. Because these minute adjustments are far too small to provide any meaningful thermal relief or confer a clear energetic advantage, we do not consider them to be biologically relevant adaptive responses to the heat stress.

**Table S9: Sensitivity analyses of parrotfishes behavioral responses across varying temporal windows and baseline approaches.** The table presents model outcomes (p-values and effect sizes) for overall mean and diel analyses, testing alternative tracking windows (1.5× and 3× MHW duration) and an isolated 1-week window excluding temporally proximate events. Significant p-values in alternative models correspond to negligible absolute effect sizes, lacking biological relevance for thermal mitigation.

| **Baseline Approach** | **Analysis Type** | **Temporal Window** | **Response Variable** | **p-value** | **Effect Size & Comments** |
| --- | --- | --- | --- | --- | --- |
| Fixed | Overall Mean | 1.5 x MHW duration | Activity | 0.73 | - |
|  |  |  | Depth | 0.65 | - |
|  |  |  | Maximum displacement | 0.11 | - |
|  |  | 3 x MHW duration | Activity | 0.76 | - |
|  |  |  | Depth | 0.72 | - |
|  |  |  | Maximum displacement | 0.12 | - |
|  |  | 1-week (excluding overlap) | Activity | 0.77 | - |
|  |  |  | Depth | 0.47 | - |
|  |  |  | Maximum displacement | 0.21 | - |
|  | Diel analysis | 1.5 x MHW duration | Activity | 0.04 | Increase of 0.1 m/s² in the 'after' period |
|  |  |  | Depth | < 0.001 | Depth reduction of 0.08m & 0.2m in the 'after' and 'MHW' periods, respectively |
|  |  | 3 x MHW duration | Activity | NA | Base model (Model 0) was the best; excluding MHW stage |
|  |  |  | Depth | < 0.001 | Depth reduction of 0.4m & 0.22m in the 'after' and 'MHW' periods, respectively |
|  |  | 1-week (excluding overlap) | Activity | 0.5 | - |
|  |  |  | Depth | 0.01 | Increase of 0.06m in depth in the 'MHW' period |
| Detrended | Overall Mean | 1.5 x MHW duration | Activity | 0.23 | - |
|  |  |  | Depth | < 0.01 | - |
|  |  | 3 x MHW duration | Activity | 0.74 | - |
|  |  |  | Depth | < 0.01 | - |
|  | Diel analysis | 1.5 x MHW duration | Activity | 0.01 | Increase of 0.05m in depth in the 'MHW' period |
|  |  | 3 x MHW duration | Activity | 0.09 | - |

### Individual behavioral anomalies

We documented an isolated mortality event following MHW 9 (an 18-day event) within the fixed baseline definition. Fish ID 1255806 exhibited a permanent drop in activity to near-zero levels shortly after the heatwave concluded (Fig. S12). While this sustained cessation of movement clearly indicates mortality, it cannot be definitively attributed to thermal stress. The individual's detection record was highly fragmented, characterized by a lack of continuous tracking prior to the event and intermittent, patchy detections throughout the MHW itself. Consequently, we report this simply as an isolated observational anomaly rather than confirmed MHW-induced mortality.

**Figure S12.** **Continuous activity profile for fish ID 1255806 associated with MHW #9.** Shaded background regions denote the defined MHW stages: 'before' (green), 'during' (red; 06/09/2019 - 24/09/2019), and 'after' (blue).

A second isolated behavioral anomaly involved fish ID 1255815, which exhibited a profound depth shift shortly after detrended MHW 2 (28/04/2018 – 07/05/2018; a 15-day event). During the post-MHW period, depth readings for this individual abruptly descended and plateaued at 38.05 m, representing the maximum operational limit of the transmitter's pressure sensor (Fig. S13a). While the fish appeared to maintain typical activity levels initially (Fig. S13b), the final transmission from this individual was received on June 9, 2018. Although a few shallower depth readings were recorded immediately after the initial descent, these sparse values may reflect the progressive onset of a technical sensor malfunction rather than true vertical movements, occurring just before the sensor ultimately froze at its maximum limit. Given these confounding factors, a definitive distinction between a delayed behavioral shift and a gradual mechanical failure cannot be conclusively resolved.

**Figure S13. Continuous depth (a) and activity (b) profiles for fish ID 1255815 associated with detrended MHW #2.** Shaded background regions denote the defined MHW stages: 'before' (green), 'during' (red), and 'after' (blue).

### Supplementary references

1.	Ørsted M, Jørgensen LB, Hůla P, Overgaard J. 2026 Integrating physiological rates of thermal stress and repair predicts heat failure during temperature fluctuations. *Ecol. Lett.* **29**, e70398.

2.	Ma C-S, Wang L, Zhang W, Rudolf VHW. 2018 Resolving biological impacts of multiple heat waves: interaction of hot and recovery days. *Oikos* **127**, 622–633.

3.	Leung JYS, Connell SD, Russell BD. 2017 Heatwaves diminish the survival of a subtidal gastropod through reduction in energy budget and depletion of energy reserves. *Sci. Rep.* **7**, 17688.
